# Cancer metabolic reprogramming and precision medicine-current perspective

**DOI:** 10.3389/fphar.2024.1450441

**Published:** 2024-10-17

**Authors:** Tingting Gao, Liuxin Yang, Yali Zhang, Ousman Bajinka, Xingxing Yuan

**Affiliations:** ^1^ Department of Gastroenterology, Heilongjiang Academy of Traditional Chinese Medicine, Harbin, China; ^2^ First Clinical Medical College, Heilongjiang University of Chinese Medicine, Harbin, China; ^3^ School of Medicine and Allied Health Sciences, University of The Gambia, Banjul, Gambia

**Keywords:** metabolic reprogramming, precision medicine, treatment strategies, immune cell, mechanism

## Abstract

Despite the advanced technologies and global attention on cancer treatment strategies, cancer continues to claim lives and adversely affects socio-economic development. Although combination therapies were anticipated to eradicate this disease, the resilient and restorative nature of cancers allows them to proliferate at the expense of host immune cells energetically. This proliferation is driven by metabolic profiles specific to the cancer type and the patient. An emerging field is exploring the metabolic reprogramming (MR) of cancers to predict effective treatments. This mini-review discusses the recent advancements in cancer MR that have contributed to predictive, preventive, and precision medicine. Current perspectives on the mechanisms of various cancer types and prospects for MR and personalized cancer medicine are essential for optimizing metabolic outputs necessary for personalized treatments.

## Introduction

**FIGURE 1:**
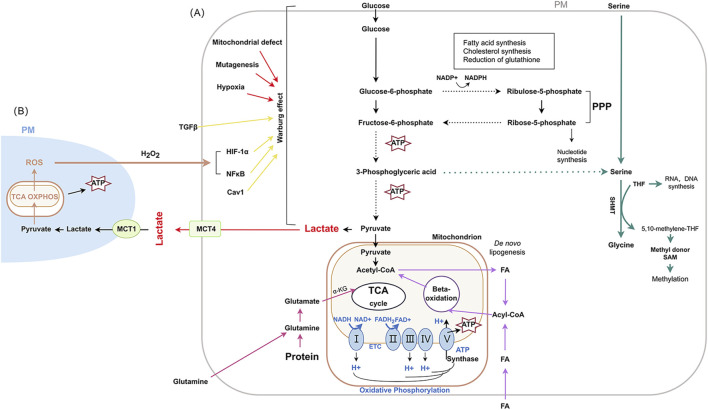
The main metabolic pathways and changes in cancer Abbreviation used: PM: plasma membrane; ROS: reactive oxygen species; TCA: tricarboxylic acid; OXPHOS: oxidative phosphorylation; ATP: adenosine triphosphate; MCT1: monocarboxylate transporter-1; TGF-β:Transforming growth factor-β; MCT4: monocarboxylate transporters 4; HIF-1α: hypoxia-inducible factor-1 α; NF-κB: nuclear factor κB; Cav1: Caveolin-1; PPP: pentose phosphate pathway; SHMT: serine hydroxy methyltransferase; THF: tetrahydrofolate; SAM: S-adenosylmethionine; α-KG: α-ketoglutarate; NADH: nicotinamide adenine dinucleotide; FA: fatty acid. FAD: flavin adenine dinucleotide; FADH2: reduced flavin adenine dinucleotide; I: NADH-coenzyme Q reductase; II: succinate coenzyme Q reductase; III: coenzyme Q cytochrome c reductase; IV: cytochrome c oxidase; V: ATP synthase.

Cancer is a multifaceted genetic disease that arises from elaborate changes to the genome. Metabolic reprogramming (MR) is considered to be one of the emerging hallmarkers of cancer, which promotes cell survival and infinite proliferation of malignant cells through changes in the characteristics of metabolic enzymes, upstream regulatory molecules and downstream metabolites (Ciardiello et al., 2022). Owing to the high metabolic plasticity, tumor cells exhibit complex metabolic patterns, with glucose metabolism, amino acid metabolism and lipid metabolism being dominant (Xu X. et al., 2023). Glucose metabolism includes glycolysis and glucose oxidative phosphorylation (OXPHOS), lipid metabolism mainly consists of fatty acid oxidation, fatty acid synthesis and cholesterol esterification, and amino acid metabolism includes pentose phosphate pathway (PPP) and serine/glycine pathway (An et al., 2024; Liu X. et al., 2024; Yang K. et al., 2023). Figure 1. In addition, there is metabolic crosstalk among glucose, lipid and amino acid metabolism. Furthermore, MR also elucidates the inherent fragility of cancer therapeutics, and the foundation of precision medicine for cancers based on MR requires detailed insights into both tumorigenesis and progression.

The challenges of drug resistance and immune evasion remain major, persistent, unsolved issues in conventional cancer treatments, and are closely related to MR and epigenetics factors (Sun et al., 2022). Epigenetic modifications play a crucial role in maintaining normal physiological functions of the body, including covalent modification of bases in DNA, post-translational modification of the terminal amino acids of histones, chromatin remodeling, and modification and regulation of non-coding RNA. A large number of studies have shown that abnormal epigenetic modifications can affect the occurrence and development of cancer by regulating cancer MR (Wang et al., 2022; Yang J. et al., 2023; Yu et al., 2018). The complex interactions between genes and the environment as epigenetic factors make it impossible to adopt one-size-fits-all approach, thus necessitating precision medicine interventions for cancer (Rulten et al., 2023). MR and dysregulation are fundamental processes that result in phenotypic alterations through molecular mechanisms, which are critical for precision therapeutic interventions for cancer. Therefore, targeting cancer metabolic pathways is a promising approach to combat drug resistance. Moreover, metabolic, epigenetic, and transcriptional regulation involving immune cell plasticity in cancers are crucial for personalized treatment (Yuan et al., 2024). Considering the diverse MR, immune evasion strategies, and drug resistance mechanisms exhibited by different cancers, it is crucial to identify a range of cancer-specific genes for biomarker screening (Wamsley et al., 2023). Therefore, this mini-review collects evidence on energy rewiring mechanisms and the prospects of personalized medicine for various cancers.

## Metabolic reprogramming and tumor microenvironment

The secondary objective of cancer MR is to reconfigure the tumor microenvironment (TME), which is primarily characterized by insufficient nutrient supply, hypoxia, and acidosis (Safi et al., 2023). The TME comprises intense interactions among tumor cells, stroma, and immune cells, which are crucial in tumorigenesis, metastasis, and drug resistance. The heterogeneity of TME not only provides survival and adaptability advantages for tumor cells, but also endows tumor cells with different reactivity and resistance to chemotherapy and targeted therapy. Immune cells also undergo MR in TME to produce metabolic adaptations associated with tolerance phenotypes, thereby exerting anti-tumor and gaining the ability to evade immune surveillance (Liu C. et al., 2024). Immunoediting intricately shapes the immunogenic profile of tumor cells and effectively undermines host anti-tumor responses throughout the process of tumorigenesis. Although intrinsic factors and metabolites regulate these processes within the TME, the interaction of immune cells, metabolites, and their niche is essential for effective treatment regimens (Buck et al., 2017). Recent findings have correlated deregulated bioenergetics programs with immunoediting, creating a counterbalance between infiltrating T cells and tumor in the TME (Tsai et al., 2023). Given the wide heterogeneity and adaptability of tumors, the TME also provide potential mechanistic targets, including oncometabolites and epigenetic modifiers (Das et al., 2024).

## Various metabolic mechanisms on cancer precision medicine

The dissemination and metastasis of cancer are driven by epithelial-to-mesenchymal transition (EMT) through invasion and migration. This process is intimately linked to MR arising from the rewiring of cellular states and signaling pathways for survival in the context of dietary deprivation. The binding of activating transcription factor 4 (ATF4) to enhancers of mesenchymal factors and amino acid deprivation-responsive genes promotes the loss of epithelial characteristics and the acquisition of transforming growth factor β (TGF-β)-signaling-associated mesenchymal traits, further enhancing lung cancer cell metastasis (Lin et al., 2024). Hence, the specific epithelial enhancers regulation of EMT through MR represents a promising intervention for precision medicine. Hypoxia profoundly impacts the TME, leading to therapy resistance. It is practicable to employ hypoxia biomarkers as predictive and prognostic indicators for solid tumors treatment (Bigos et al., 2024). Lipid metabolism holds potential in targeting isocitrate dehydrogenase 1 (IDH1) and IDH2 for metabolic interventions. Distinct fatty acid metabolism is detected between IDH1 and IDH2, making these mutated genes a potential focus for precision medicine (Thomas et al., 2023). In the targeted therapy of cancer, regulators such as hexokinase 2 (HK2), pyruvate kinase M2 (PKM2), enolase 1 (ENO1), and lactate dehydrogenase A (LDHA) are crucial for the glycolytic pathway. At the transcriptional level, glycolysis is regulated by p53, c-Myc, hypoxia-inducible factor 1 (HIF1), and sine oculis homeobox homolog 1 (SIX1). Additionally, post-translational modifications such as methylation, phosphorylation, ubiquitination, and acetylation also exert significant roles in signal transduction and MR within the glycolytic pathway (Ni et al., 2024).

The response of individual patients to cisplatin treatment have revealed metabolic changes as factors influencing sensitivity and resistance, highlighting the need for personalized medicine for cisplatin resistance and other cancer resistant therapies (Yu et al., 2023). Combination therapy is essential for glioblastoma, and epidermal growth factor receptor (EGFR)-activated MR holds great potential. Temozolomide’s antitumor effects, targeting the mevalonate and EGFR/AKT pathways, demonstrate significant clinical management of glioblastoma (Cui et al., 2023). In glioblastoma multiforme, the uptake of phospholipids and fatty acid synthesis is increased, while glycerolipid and glycerophospholipid metabolism is aberrant, disclosing its distinctive metabolic characteristics (Miao et al., 2023). A significant breakthrough has been achieved in the application of predictive medicine for gliomas, where features related to mitochondrial genome composition can predict its sensitivity to chemotherapy drugs, thereby exerting a positive impact on the prognosis of patients (Wu J. et al., 2023).

Luminal B breast cancer (LBBC) has a complex molecular landscape, and a comprehensive muti-platform analysis offers valuable targets and signaling pathways for the examination of differences between the two subtypes to attain more precise treatment of LBBC (Wang H. et al., 2023). Phosphoinositide 3-kinase (PI3K) inhibitors combined with autophagy have a strong rationale for breast cancer treatment. For instance, the metabolism of breast cancer is influenced by mitochondrial translation dysregulation and loss of core binding factor subunit β (CBFB) function (Malik et al., 2023). A metabolic switch involving triple-negative breast cancer (TNBC) shifts from glycolysis to fatty acid β-oxidation (FAO) through the inhibition of PKM2. Impaired histone methyltransferase, enhancer of zeste homolog 2 (EZH2), can be recruited to solute carrier family 16 member 9 (SLC16A9), a carnitine transporter, coordinated by the direct interaction of PKM2, thus epigenetically influencing tumor progression (Zhang Y. et al., 2024). In cervical squamous cell carcinoma (CESC), lipid metabolism-related genes (LMRGs) signature plays a significant role in advancing precision medicine strategies for the management of patients with cervical cancer by enhancing CESC prognostication (Wang and Zhang, 2024). The depletion of aconitate decarboxylase 1 (ACOD1) has been shown to reduce the levels of the immune metabolite itaconate, while simultaneously driving macrophages to polarize strongly and persistently towards a pro-inflammatory state, demonstrating an enhanced tumour-inhibiting ability and thereby improving ovarian cancer (OC) survival (Wang X. et al., 2023).

Overexpression of mucin 1 (MUC1) can promote cancer cell proliferation by regulating cell metabolism, and tumor-related MUC1 exhibiting loss of apical localization and aberrant glycosylation in kidney cancer, especially in renal cell carcinoma (RCC) (Milella et al., 2024). Fatty acid metabolism presents the potential clinical application value of PD-1/PD-L1 in the TME of clear cell renal cell carcinoma (ccRCC), thereby providing a predictive treatment response for personalized medicine (Lin et al., 2023). Regulating glycolytic metabolism through targeting the c-Myc oncoprotein is demonstrated in the ubiquitin specific peptidase 43 (USP43) enzyme in bladder cancer. The degradation of c-Myc is achieved through interference with f-box and WD repeat domain containing 7 (FBXW7) by USP43 upregulation (Li M. et al., 2024).

In liver cancer, the identification and characterization of novel invasive cancer types will enhance the understanding of invasion and metastasis, leading to the development of novel precision therapies (Wu L. et al., 2023). In hepatocellular carcinoma (HCC), solute carrier family 25 member 15 (SLC25A15) is hypoxia-responsive with low glutamine reprogramming, facilitating anti-PD-L1 therapy (Zhang Q. et al., 2024). The loss of NADPH oxidase 4 (NOX4) in HCC induces MR in a nuclear factor erythroid 2-related factor 2 (Nrf2)/Myc-dependent manner, promoting tumor progression and making this tumor suppressor function a targeted therapy (Penuelas-Haro et al., 2023). Recently, nanoparticles-mediated co-delivery of cofilin 1 (CFL1) silencing with sorafenib, a chemotherapeutic agent, showed elevated inhibitory properties for HCC tumor growth without exhibiting significant toxicity (Li S. et al., 2023).

For Lung cancer, solute carrier family 3 member 2 (SLC3A2) acts as a metabolic switch factor in tumor-associated macrophages (TAM), suggesting that lung adenocarcinoma (LUAD) phenotyping reprogramming occurs via arachidonic acid (Li Z. et al., 2023). Oncogenic mutations are likely to facilitate MR within cancer cells for sustaining their energy and biomass demands. Results obtained from non-small cell lung cancer (NSCLC) cells with varying EGFR and kristen rat sarcoma (KRAS) statuses indicated that NSCLC cell lines possess a heterogeneous metabolic profile, which could facilitate metabolically targeted therapy for NSCLC patients through identification and stratification (Mendes et al., 2023).

Acute myeloid leukemia (AML) demonstrates a resistance to immunosurveillance and chemotherapy in leukemia stem cells (LSCs). For instance, inhibition of the Src homology region 2 (SH-2) domain-containing phosphatase 1 (Shp1) can alter the energy mechanisms of LSCs to confer sensitivity to chemotherapeutic drugs and presenting a promising prospect for surmounting resistance in AML (Xu et al., 2024). Through the AKT-β-catenin pathway, the expression of phosphofructokinase platelet (PFKP) is upregulated, which increased the metabolic activities and promote the degradation of Myc, thereby reducing the abilities of LSCs to evade the immune system and enhance the sensitivity to chemotherapy. Despite the emergence of drug resistance, precision treatment with a combined synergistic effect of mTOR and fibroblast growth factor receptor 1 (FGFR1) inhibitors is promising for T-cell acute lymphoblastic leukemia (T-ALL). This is practically achievable through MR, leading to the reversal of FGFR1 inhibitor resistance (Zhang et al., 2023). In osteosarcoma, clustering of lactic acid metabolism could identify the NADH dehydrogenase (ubiquinone) complex I assembly factor 6 (NDUFAF6, also known as C8ORF38) gene, which is a lactic acid metabolism-related gene and a prognostic marker, thus making this gene a functional target for individuals with this cancer type (Wang et al., 2024a).

**FIGURE 2:**
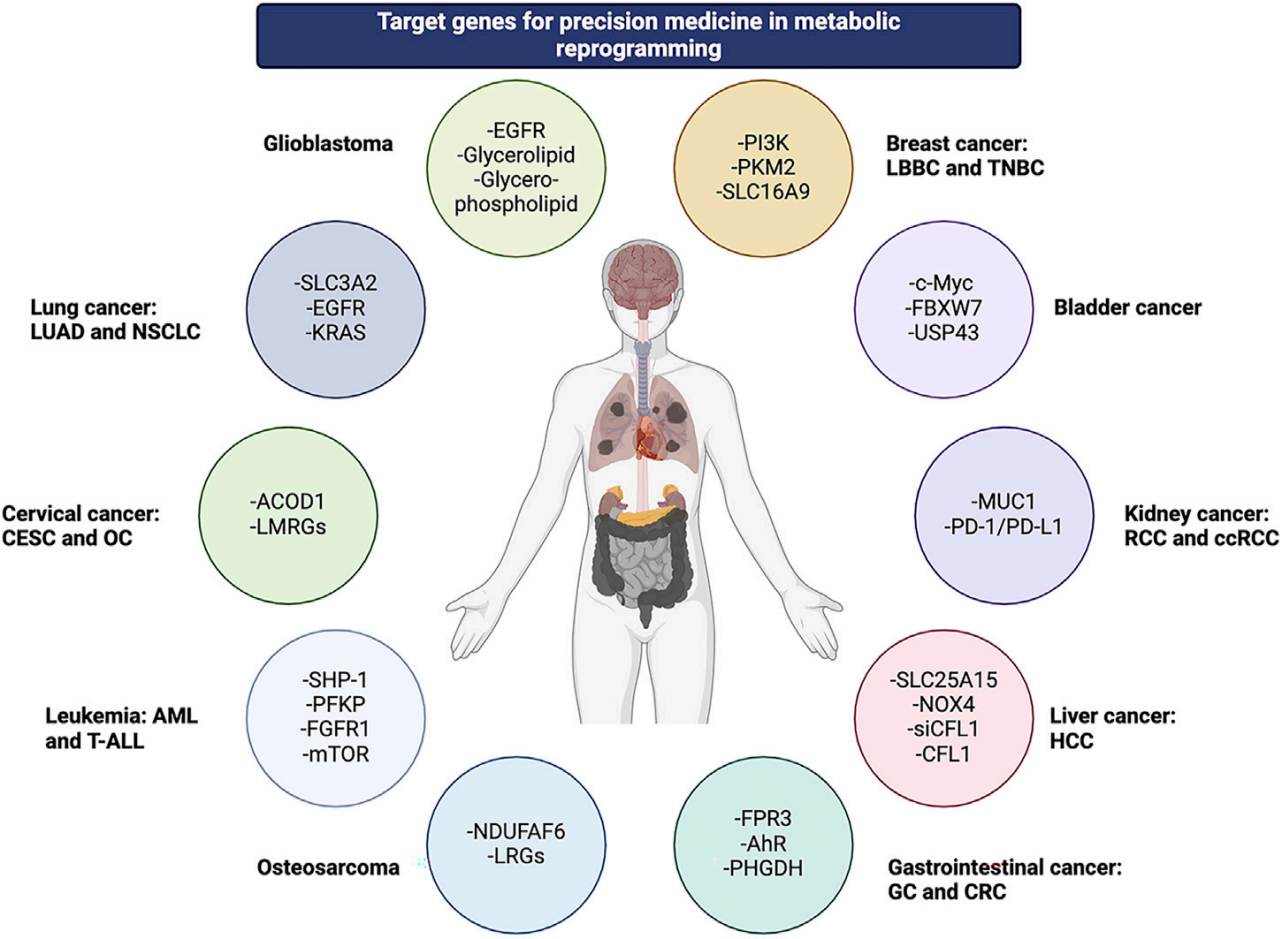
Various genes across multiple cancer types are involved in metabolic reprogramming as potential targets for precision medicine.

While promising for metabolism-based therapies, advanced feature analysis through single-cell, multi-omics and spatial technologies, as well as accurate tracking of dynamic changes in metabolic adaptation, will promote the application of precision metabolic therapy in cancer treatment (Wu Z. et al., 2023). Trans-omics, as part of the quest for multiple biomarkers, provides reliability and accuracy for cancer diagnosis. With growing interest in lung cancer and MR, specific metabolites such as lipids can offer a comprehensive analysis of lung cancer. To some extent, precision therapy for lung cancer depends on this novel trans-omics network approach (Yan et al., 2024). Considering the impact of MR on inter-patient heterogeneity, a model pipeline has been developed for ensemble learning, providing insights for the treatment of LUAD (Sun et al., 2024). Formyl peptide receptor 3 (FPR3) can hinder the nuclear translocation of the nuclear factor of activated T cells 1 (NFATc1) by blocking cytoplasmic calcium influx and deactivating the NFATc1-binding neurogenic locus notch homolog protein 3 (NOTCH3) promoter. This process leads to the downregulation of glycolysis through the blocking of the AKT/mTORC1 signaling pathway and NOTCH3 expression. Gastric cancer precision therapy has proven the role of FPR3 in a calcium-dependent manner, thus providing insights into other precision therapy options (Wang et al., 2024b). Cancer-associated fibroblasts (CAF) have metabotropic subtypes with promising precision therapy applications, especially in liver metastases of colorectal cancer (CRC). The interaction of different immune cells with various communication strategies determines the sensitivity of chemotherapeutic drugs (Wu C. et al., 2023). Phosphoglycerate dehydrogenase (PHGDH) can modulate aryl hydrocarbon receptor (AhR) signaling and the redox-dependent autophagy pathway in CRC. Consequently, combination therapy that includes inhibition of both AhR and PHGDH is promising for this type of cancer (Li HM. et al., 2024). Additionally, a notable correlation exists between the expression level of PD-L1 and the individualized treatment regimen, as demonstrated by the mitochondrial pyruvate carrier 3 (MPC3) energy type in the treatment of esophageal squamous cell carcinoma (ESCC) (Wang Z. et al., 2024). Figure 2.

## Prospects on metabolic reprogramming and cancer personalized medicine

A model derived from machine learning could potentially identify the metabolic landscape of gastric cancer for early detection (Chen et al., 2024). Another promising approach involves ultrasound-mediated cancer diagnosis using noninvasive mass spectrometry imaging (MSI), particularly effective for breast cancers, offering detailed “fingerprints” of elasticity and histopathology metabolism (Zhou et al., 2024). MR can impact T cell exhaustion via R-loop score patterns present tailored treatment avenues, crucial as R-loop regulators drive tumor progression (Zhang S. et al., 2024). Moreover, MR modulates tumor cell survival under hypoxia, influencing tumor proliferation and genomic stability (Abou Khouzam et al., 2023). The hypoxic TME also enhances chimeric antigen receptor (CAR)-T cell efficacy as a durable antitumor strategy (Zhu et al., 2024).

The risk of cancer, notably metabolic cancers linked to obesity, affects the phenotype and metabolism of immune cells. MR studies focusing on metabolic disorder-induced cancers may uncover biomarkers crucial for immunotherapy (Rosario et al., 2023). Oxidative stress and metabolism-related genes serve as reliable predictors for personalized medicine, aiding in targeted drug therapies and early diagnosis of patients at high risk for stomach adenocarcinoma (STAD) (Dong et al., 2023). In melanoma, targeted therapies exploiting metabolic phenotypes have shown promise with immune checkpoint inhibitors (ICI) (Carlino et al., 2021). Additionally, the study of platelet-derived microparticles in chronic lymphocytic leukemia (CLL) has revealed potential for precision medicine by highlighting immunologically dysfunctional B-lymphocytes (Gharib et al., 2023). A347D mutant p53 variant is implicated in cancer susceptibility among individuals with Li-Fraumeni syndrome (Choe et al., 2023). Mitochondrial OXPHOS adversely impacts antitumor immunity by reducing tumor-infiltrating T cells (Zhao et al., 2022). Future research should explore tumor heterogeneity within the TME and its implications for immune responses in precision medicine (Xu W. et al., 2023).

Improving cancer treatment outcomes and enhancing precision and predictive medicine require MR to reveal treatment vulnerabilities. As one of the newest approaches, some metabolic molecules have already progressed from the preclinical stage to the later stage of clinical trials. For example, AG-120 (ivosidenib), an inhibitor of the IDH1 mutant enzyme, has an acceptable safety profile and clinical activity, according to preliminary data from a Phase I clinical trial recruiting cancer patients with the IDH1 mutation (Popovici-Muller et al., 2018). Inhibition of fatty acid synthase (FASN) revealed a distinct tumor response, which were exacerbated by proliferative potential and mitochondrial respiration. The differences in expression patterns exhibited by FASN in pancreatic ductal adenocarcinoma (PDAC) illuminate the hallmarks of lipid metabolism (Chianese et al., 2023). TVB-2640, a FASN inhibitor, was found to be a well-tolerated oral agent in a Phase II study of recurrent high-grade astrocytoma and could be safely combined with bevacizumab to improve progression-free survival (PFS) (Kelly et al., 2023). Preclinical and clinical trial data suggest that the combination of CB-839, a glutaminase inhibitor, and capecitabine can be an effective treatment for PIK3CA-mutated CRC (Grkovski et al., 2020). CPI-613 is a novel anticancer agent that selectively targets altered forms of mitochondrial energy metabolism in tumor cells, causing changes in mitochondrial enzyme activity and REDOX status, which lead to apoptosis, necrosis, and autophagy in tumor cells. The use of CPI-613 in combination with modified FOLFIRINOX in patients with metastatic pancreatic cancer requires validation in a phase 2 trial (Alistar et al., 2017). However, despite the outstanding results of basic research in tumor MR, many metabolic enzymes have been targeted as tumor therapy, but the vulnerability of specific tumor types to specific inhibitors remains to be further investigated, whether it is a single drug or combination chemotherapy, radiotherapy, targeted therapy or immunotherapy.

In summary, the concept of modifying cancer cell metabolism to slow disease progression while enhancing immune cell function represents a groundbreaking approach in personalized MR intervention. Essential to this endeavor are enzyme inhibitors, metabolic enzyme modifications, pathway interactions, MR drug delivery targets, and methodical study designs (Wang Q. et al., 2024). The efficacy of MR is contingent upon a comprehensive understanding of the diverse metabolic environments present within cancers, which influence DNA repair mechanisms and therapeutic resistance, thus elucidating the broader metabolic landscape in cancer (Das et al., 2023). A novel therapeutic strategy that targets the interplay between cancer epigenetics, metabolism, and DNA repair pathways holds promise (Xing et al., 2023). Recent advances in molecular subtyping, including proteomic, genomic, transcriptomic, and phosphoproteomic profiling, along with assessments of microenvironment dysregulation, genetic alterations, and kinase-substrate regulatory networks, are poised to yield distinct therapeutic responses.

## Conclusion

Personalized medicine in cancer therapy is patient-dependent and largely influenced by gene profile. Cancer cells survive within the TME through mechanisms of energy reprogramming. Given the genetic predisposition to various metabolic disorders, research into personalized pharmacotherapy will enhance the long-term efficacy of anti-cancer agents and mitigate drug resistance. Cancer precision medicine based on MR to suppress cancer growth while enhancing immunity is part of the hallmark of cancer research. While this mini review gives some examples of the forms of cancer with distinct energy requirements, obstructing the sources of these energies to rewire metabolic output will give essential requirements for personalized medicine.

## Glossary

ACOD1: Aconitate decarboxylase 1
AhR: Aryl hydrocarbon receptor
AML: Acute myeloid leukemia
ATF4: Activating transcription factor 4
CAF: Cancer-associated fibroblasts
CAR: Chimeric antigen receptor
CBFB: Core binding factor subunit β
ccRCC: Clear cell renal cell carcinoma
CESC: Cervical squamous cell carcinoma
CFL1: Co-delivery of cofilin 1
CLL: Chronic lymphocytic leukemia
CRC: Colorectal cancer
EGFR: Epidermal growth factor receptor
EMT: Epithelial-to-mesenchymal transition
ENO1: Enolase 1
ESCC: Esophageal squamous cell carcinoma
EZH2: Enhancer of zeste homolog 2
FASN: Fatty acid synthase
FAO: Fatty acid β-oxidation
FBXW7: F-box and WD repeat domain containing 7
FGFR1: Fibroblast growth factor receptor 1
FPR3: Formyl peptide receptor 3
HCC: Hepatocellular carcinoma
HIF1: Hypoxia-inducible factor 1
HK2: Hexokinase 2
IDH1: Isocitrate dehydrogenase 1
KRAS: Kristen rat sarcoma
LBBC: Luminal B breast cancer
LDHA: Lactate dehydrogenase A
LMRGs: Lipid metabolism-related genes
LSCs: Leukemia stem cells
LUAD: Lung adenocarcinoma
MPC3: Mitochondrial pyruvate carrier 3
MR: Metabolic reprogramming
MSI: Mass spectrometry imaging
MUC1: Mucin 1
NFATc1: Nuclear factor of activated T cells 1
NOX4: NADPH oxidase 4
NOTCH3: Notch homolog protein 3
NDUFAF6: NADH dehydrogenase (ubiquinone) complex I assembly factor 6
Nrf2: Nuclear factor erythroid 2-related factor 2
NSCLC: Non-small cell lung cancer
OXPHOS: Oxidative phosphorylation
OC: Ovarian cancer
PDAC: Pancreatic ductal adenocarcinoma
PHGDH: Phosphoglycerate dehydrogenase
PKM2: Pyruvate kinase M2
PPP: Pentose phosphate pathway
PI3K: Phosphoinositide 3-kinase
PFS: Progression-free survival
PFKP: Phosphofructokinase platelet
RCC: Renal cell carcinoma
SH-2: Src homology region 2
Shp1: Phosphatase 1
SIX1: Sine oculis homeobox homolog 1
SLC3A2: Solute carrier family 3 member 2
SLC25A15: Solute carrier family 25 member 15
SLC16A9: Solute carrier family 16 member 9
STAD: Stomach adenocarcinoma
TAM: Tumor-associated macrophages
T-ALL: T-cell acute lymphoblastic leukemia
TGF-β: Transforming growth factor β
TME: Tumor microenvironment
TNBC: Triple-negative breast cancer
MPC3: Mitochondrial pyruvate carrier 3
USP43: Ubiquitin specific peptidase 43
